# Envy

**Published:** 1878-02-20

**Authors:** 


### ENVY.

It is a lamentable fact that medical men in the same community are more frequently unfriendly and envious of each other than men of other professions. A common source of these troubles between medical gentlemen, we think, may be found in the indiscreet conduct of over-zealous friends, who often express opinions and convey reports which are derogatory to rival practitioners. Medical men should not encourage but discountenance adverse reports touching the character or professional acquirements of medical brethren. For no matter how agreeable to one's vanity to hear disparaging reports of his rival's skill as compared with his own in the end, it is certain that no good will result to either party from these petty dissensions and jealousies. The physician's calling is a noble one, and ought to develop noble and generous impulses as between those who practice it. It were well that the physicians in every community would form medical societies, and come together frequently in free and friendly intercourse, talk over their troubles, report their views and experience, and resolve to sustain and encourage each other in all the social and professional relations of life.

We will go even farther than will perhaps be justified by many who endorse what is said above, and that is, that even those of different schools of practice need not quarrel. While consultations may not be admissible by reason of radical and irreconcilable differences of opinion, yet, it is not inconsistent with the honor of the true physician to tolerate these differences kindly, and to treat with courtesy those who practice systems different from his own.

We here make no assault upon true ethics, but we desire to inculcate liberal and generous sentiments among all those who practice the profession of medicine.

